# Pulmonary Cellular Toxicity in Alpha-1 Antitrypsin Deficiency

**DOI:** 10.1016/j.chest.2024.02.013

**Published:** 2024-02-14

**Authors:** Kristine M. Abo, Carly Merritt, Maria C. Basil, Susan M. Lin, Edward Cantu, Michael P. Morley, Pushpinder Bawa, Marissa Gallagher, Derek E. Byers, Edward E. Morrisey, Andrew A. Wilson

**Affiliations:** a Center for Regenerative Medicine of Boston University and Boston Medical Center, Boston, MA; b The Pulmonary Center and Department of Medicine, Boston University School of Medicine, Boston, MA; c Department of Medicine, Perelman School of Medicine, University of Pennsylvania, Philadelphia, PA; d Penn-CHOP Lung Biology Institute, Perelman School of Medicine, University of Pennsylvania, Philadelphia, PA; e Division of Cardiovascular Surgery, Department of Surgery, Perelman School of Medicine, University of Pennsylvania, Philadelphia, PA; f Division of Pulmonary and Critical Care Medicine, Washington University School of Medicine, St Louis, MO; g Department of Cell and Developmental Biology, Perelman School of Medicine, University of Pennsylvania, Philadelphia, PA

To the Editor:

Alpha-1 antitrypsin deficiency (AATD) is caused by mutations in *SERPINA1*, the gene encoding alpha-1 antitrypsin (AAT). AAT, an antiprotease with immunomodulatory functions, is an abundant serum protein secreted mainly by hepatocytes. AAT is also known to be produced by extrahepatic cells,^1^, ^2^, ^3^, ^4^ including by those that contribute to the pool present in the alveolar epithelial lining fluid.^5^ Patients with AATD who are homozygous for the Z mutation (ZZ-AATD) are susceptible to lung and liver disease. Although AATD-associated liver disease results from accumulation of misfolded, polymerized Z-AAT within hepatocytes and associated gain-of-function toxicity,^6^ AATD-associated emphysema has been attributed primarily to loss of antiprotease function, unopposed neutrophil elastase activity, and resultant pulmonary parenchymal destruction.

Protease-antiprotease imbalance alone, however, does not fully explain the pulmonary phenotype in AATD. Multiple studies have identified proinflammatory consequences of Z-AAT polymer deposition in the lung interstitium, including activation and trafficking of innate immune cells.^7^^,^^8^ In addition, antiprotease replacement via AAT augmentation therapy alone is not sufficient to fully arrest accelerated pulmonary function decline in some individuals with ZZ-AATD.^9^^,^^10^ Prior work has identified extrahepatic cells that produce AAT, including myeloid cells^1^, ^2^, ^3^^,^^11^ and airway epithelial cells.^4^ More recently, single-cell RNA sequencing data sets of adult human lung tissue have consistently demonstrated *SERPINA1* transcript expression in alveolar type 2 cells (AT2s) and macrophages.^12^, ^13^, ^14^, ^15^, ^16^ AT2s are facultative progenitors of the distal lung, the structure primarily injured in emphysema observed in AATD. Whether the human alveolar epithelium produces AAT protein in either healthy or diseased lungs, however, has not been established. Here, we report that AAT protein is expressed by human adult lung AT2s and macrophages, resulting in intrinsic cellular stress among resident lung cells in ZZ-AATD.

## Methods

### Human Lung Donors

Seventeen human lung samples used for single-cell RNA sequencing and immunofluorescence experiments in this study were obtained through an established protocol (PROPEL, University of Pennsylvania). Control donors formerly (0-2.5 pack-y) or never used tobacco; all COPD (≥ 30 pack-y) and AATD (< 30 pack-y) donors formerly used tobacco. All wild-type patients with COPD and AATD had severe obstructive disease (FEV_1_ 17%-32% predicted). Human lung tissue from an additional seven donors used for immunofluorescence studies were obtained from the Washington University School of Medicine in St. Louis. All samples were used with consent from the patient, next of kin, or health care proxy.

### Single-Cell RNA Sequencing

Distal lung parenchymal samples were dissociated and CD45-reduced as previously described.^13^ An average of 12,000 cells per donor were profiled by single-cell RNA sequencing. Donor *SERPINA1* genotype was verified by single-nucleotide polymorphism calling. Integration was performed using Harmony.^17^ Processing and downstream analyses were performed using Seurat V4.^18^ Top variable features were selected by vst, dimensionality reduction was performed by principal component analysis, and clustering was performed using the Louvain algorithm at resolution 0.5. Differential gene expression was tested using MAST.^19^ Cell identities were assigned to Louvain clusters, using previously published single-cell atlas expression signatures.^13^^,^^14^^,^^20^^,^^21^ Gene set enrichment analysis (GSEA) was performed using fgsea^22^ for Hallmark gene sets^23^ in gene lists ranked by Wilcoxon rank-sum test and area under receiver operator curve using Presto.^24^ Normalized enrichment scores for tests with Benjamini-Hochberg-adjusted *P* < .01 and false discovery rate < 0.05 were visualized using ggplot.^25^ Regulon analysis was performed using DoRothEA^26^ confidence level “A” interactions.

All raw data files as well as processed data are available for download from Gene Expression Omnibus: GSE168191 (peripheral samples) contains wild-type COPD and control sequencing data. GSE227210 contains AATD sequencing data as well as the Seurat object containing processed data and metadata. Gene sets used for cell identity assignment, code applied to generate figures, detailed methods, and an interactive web application allowing interrogation of sequencing data can be accessed via our website.^27^

### Immunofluorescence Microscopy

Peripheral human lung samples from 16 donors (4 wild-type “MM” COPD, 8 ZZ-AATD, 4 control participants without chronic lung disease) underwent staining with the following antibodies overnight: AAT (Santa Cruz sc-59438, 1:100), pro-surfactant protein C (pro-SFTPC) (Seven Hills WRAB-9337, 1:500), CD68 (Abcam Ab172730, 1:100), and CC3 (Sigma C8487, 1:100) followed by secondary antibody staining for 1 hour. Nuclei were stained with Hoechst. Images were taken at 20× magnification (quantification images) on a Nikon Ni-E fluorescent microscope or at 60× magnification (representative images) on a Leica SP5 confocal microscope. Percent co-localization was determined through manual counting of randomly chosen fields-of-view using FIJI (ImageJ).

## Results

**Figure 1:**
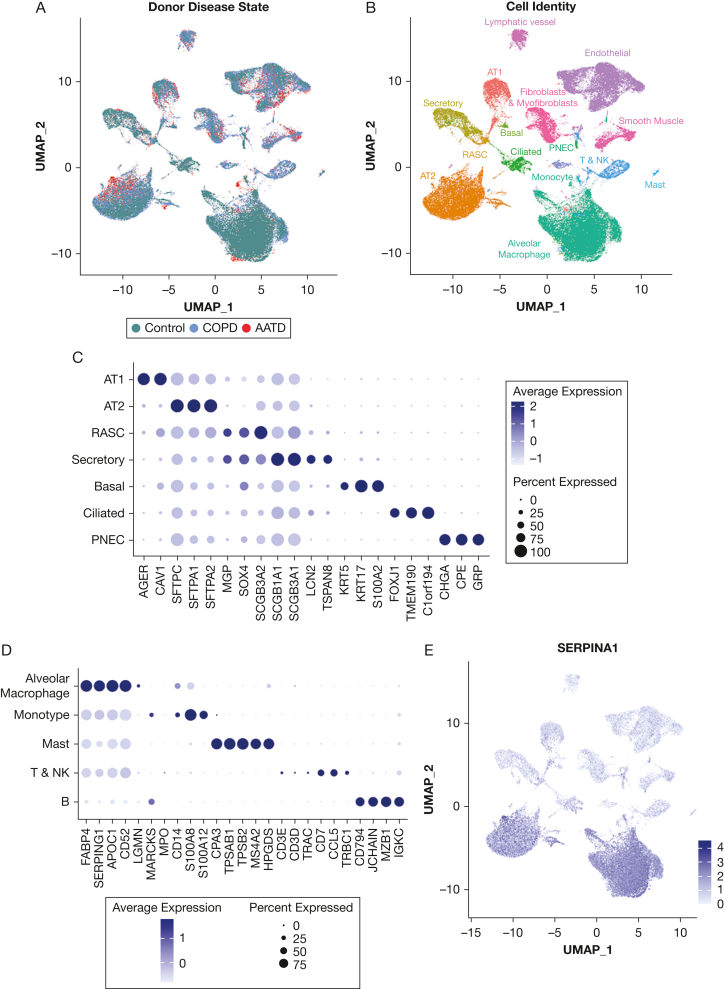

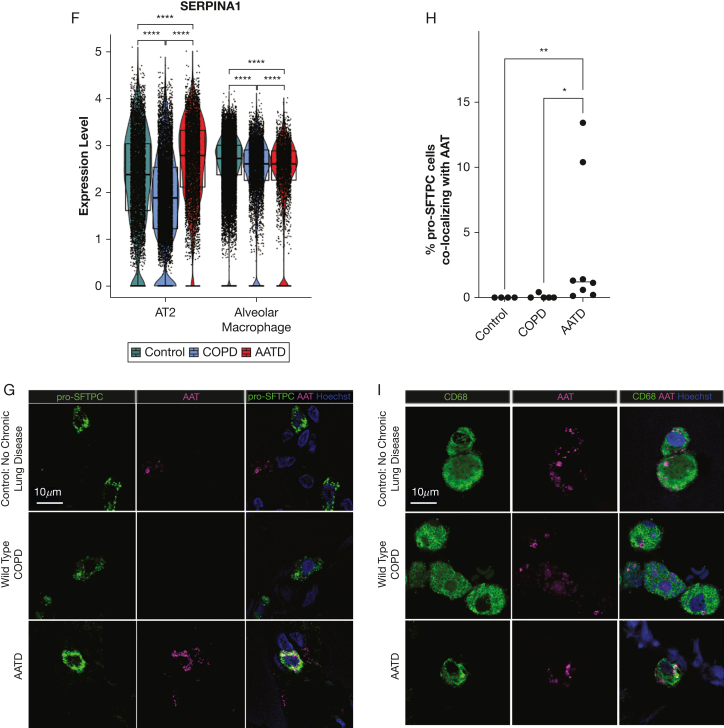
SERPINA1 and alpha-1-antitrypsin (AAT) are expressed in human alveolar type 2 cells (AT2s). (A-F) Peripheral human lung samples from 11 donors (4 wild-type “MM” COPD, 2 patients with alpha-1 antitrypsin deficiency [AATD] homozygous for the Z mutation [ZZ-AATD], and 5 healthy controls without chronic lung disease) were dissociated, CD45-reduced, and profiled by single-cell RNA sequencing. (A) Uniform manifold approximation and projection (UMAP) of peripheral human lung samples profiled by scRNA-Seq, labeled by donor disease state. (B) UMAP annotated by cell identity. (C) Dot plot of pulmonary epithelial marker gene expression among pulmonary epithelial cell clusters. (D) Dot plot of immune cell marker gene expression among immune cell clusters. (E) Feature plot of SERPINA1 expression and distribution in UMAP. (F) Violin plot of SERPINA1 expression in AT2s and alveolar macrophages and split by disease state of donor (∗∗∗∗*P* < .0001, Wilcoxon rank-sum test). (G) Representative immunofluorescence images of AAT (magenta) and pro-surfactant protein C (pro-SFTPC) (green) in distal human lung tissue from wild-type COPD, ZZ-AATD, and control donors. (H) Quantification of percentage of AAT+/SFTPC+ cells relative to the total number of pro-SFTPC+ cells (∗*P* < .05, ∗∗*P* < .01, Kruskal-Wallis test). N = 15 donors (4 wild-type COPD, 8 ZZ-AATD, 3 control). (I) Representative immunofluorescence images of AAT (magenta) and CD68 (green) in distal human lung tissue from wild-type COPD, ZZ-AATD, and control donors. AT1 = alveolar type 1 cell; PNEC = pulmonary neuroendocrine cell; RASC = respiratory airway secretory cell.

We profiled peripheral human lung samples from 11 donors (4 wild-type “MM” COPD, 2 ZZ-AATD, 5 “MM” control participants without chronic lung disease) by single-cell RNA sequencing after CD45 reduction (Fig 1A). Cell identities (Fig 1B) were assigned to Louvain clusters using previously published single-cell atlas expression signatures.^13^^,^^14^^,^^20^^,^^21^ Cells clustered by lineage rather than disease state (Fig 1B) or individual donor. We observed *SERPINA1* expression in the distal human lung in cells of myeloid lineage (macrophages and monocytes) and in AT2s across disease states (Fig 1E-F). To determine whether AT2s express AAT, we analyzed peripheral human lung tissue from a partially overlapping set of 15 donors by immunostaining for AAT and pro-SFTPC. Although AAT-producing cells rapidly secrete wild-type M-AAT, the efficiency of this process is substantially reduced for misfolded Z-AAT.^28^ Consistent with this kinetic, we identified detectable AAT in 0.1% to 13.4% of pro-SFTPC-positive cells in ZZ-AATD lung parenchyma, compared with extremely rare (wild-type COPD) or absent (non-lung disease control participants) AAT+/pro-SFTPC+ cells in non-AATD tissue (Fig 1G-H). By contrast, immunostaining for AAT and the macrophage marker CD68 identified significant co-staining across disease states (Fig 1I).

**Figure 2:**
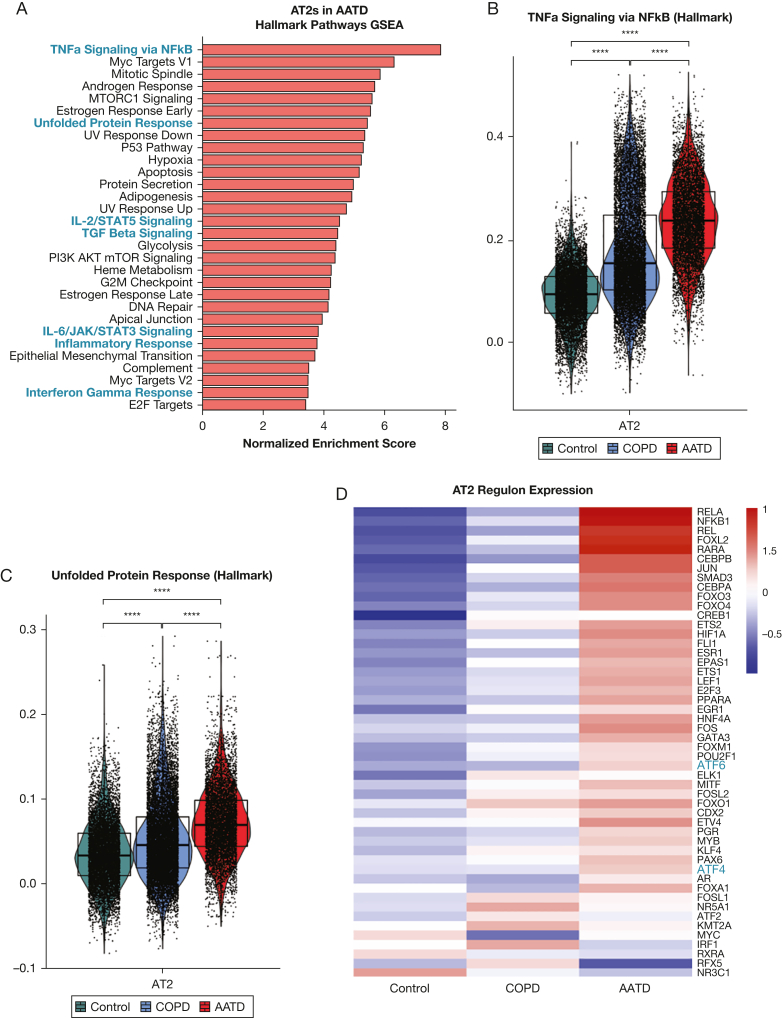

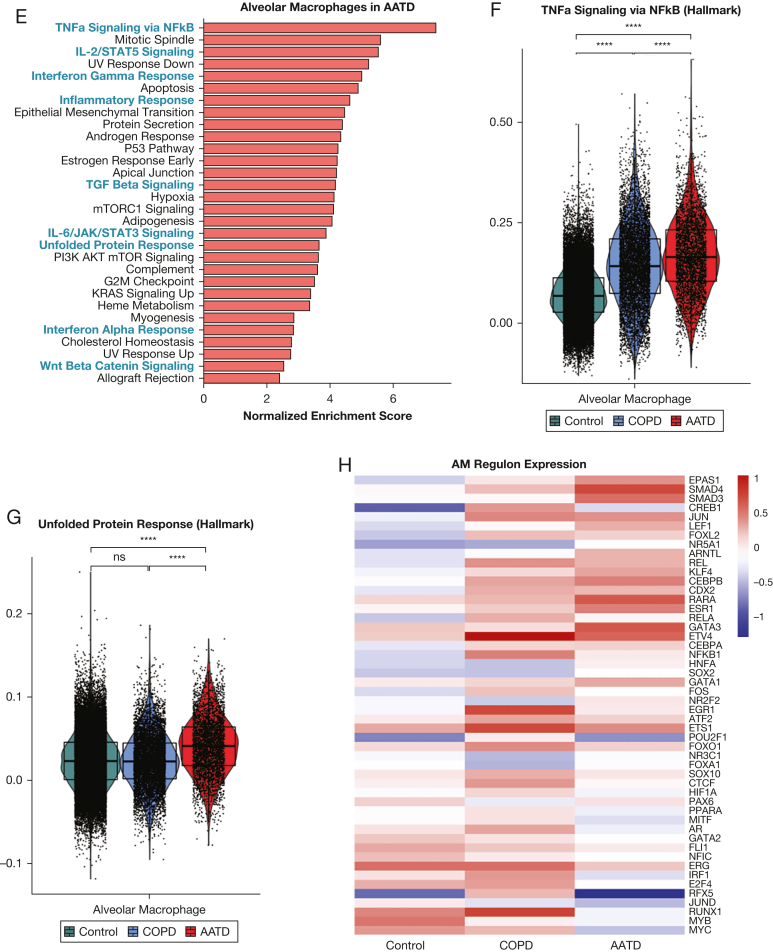

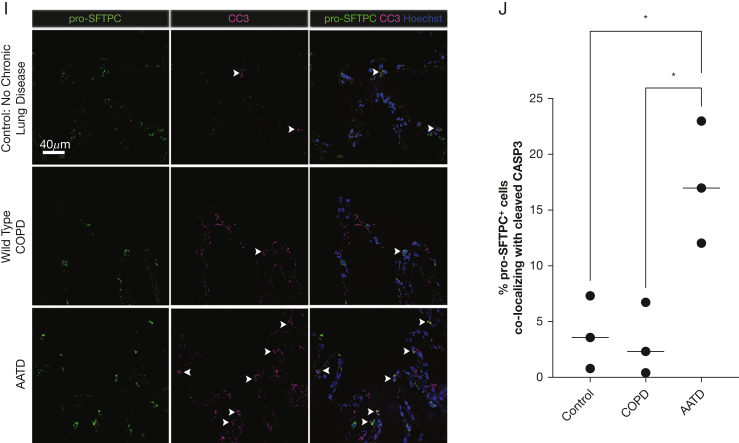
SERPINA1 expression correlates with cellular stress in alveolar type 2 cells (AT2s) and in alveolar macrophages in patients with alpha-1-antitrypsin deficiency (AATD) homozygous for the Z mutation (ZZ-AATD). (A) Gene Set Enrichment Analysis (GSEA, using Hallmark gene sets, adjusted *P* < .01, false discovery rate [FDR] < 0.05) of differentially upregulated transcripts in AT2s in AATD compared with all AT2s. Blue text identifies gene sets discussed in the text. (B) Violin plot of module score for Hallmark 2020 gene set term “TNFa Signaling via NFkB” in AT2s split by disease state of donor. (∗∗∗∗*P* < .0001, Wilcoxon rank-sum test). (C) Violin plot of module score for Hallmark 2020 gene set term “Unfolded Protein Response” in AT2s split by disease state of donor. (∗∗∗∗*P* < .0001, Wilcoxon rank-sum test). (D) Heat map of differential regulon (curated set of transcription factor target transcripts) expression in AT2s. Regulon analysis was performed for differentially expressed transcripts in AT2s in AATD compared with all AT2s using DoRothEA confidence level “A” interactions.^24^ The top 50 most variable regulons are plotted, ranked by difference between AATD and Control. (E) GSEA (using Hallmark gene sets, adjusted *P* < .01, FDR < 0.05) of differentially upregulated transcripts in alveolar macrophages in AATD compared with all alveolar macrophages. Blue text identifies gene sets discussed in the text. (F) Violin plot of module score for Hallmark 2020 gene set term “TNFa Signaling via NFkB” in alveolar macrophages split by disease state of donor. (∗∗∗∗*P* < .0001, Wilcoxon rank-sum test). (G) Violin plot of module score for Hallmark 2020 gene set term “Unfolded Protein Response” in alveolar macrophages split by disease state of donor. (∗∗∗∗*P* < .0001, Wilcoxon rank-sum test). (H) Heat map of differential regulon (curated set of transcription factor target transcripts) expression in alveolar macrophages (AM). Regulon analysis was performed for differentially expressed transcripts in AMs in AATD compared with all AMs using DoRothEA confidence level “A” interactions.^26^ Top 50 most variable regulons are plotted, ranked by difference between AATD and control. (I) Representative immunofluorescence images of cleaved caspase 3 (magenta) and pro-SFTPC (green) in distal human lung tissue from wild-type COPD, ZZ-AATD, and control/no COPD donors. (J) Quantification of CC3+/pro-SFTPC+ cells relative to the total number of pro-SFTPC+ cells (∗*P* < 0.05, one-way analysis of variance). N = 9 donors (3 wild-type COPD, 3 ZZ-AATD, 3 control/no COPD). NFkB = nuclear factor kappaB; pro-SFTPC = pro-surfactant protein C; TGF = transforming growth factor; TNF = tumor necrosis factor.

Next, we asked whether expression of mutant *SERPINA1* correlates with transcriptomic changes in ZZ-AATD. We found that in patients with ZZ-AATD, AT2s and alveolar macrophages differ transcriptomically from those in wild-type COPD and control participants without chronic lung disease. Differentially expressed genes that were upregulated in AT2s and alveolar macrophages in AATD were enriched for Hallmark gene sets associated with inflammatory signaling (“tumor necrosis factor α [TNFα] signaling via nuclear factor kappaB [NFkB]”, “IL-2/STAT5 Signaling”, “IL-6/JAK/STAT3 Signaling”) as well as the “Unfolded Protein Response” (Fig 2A-C, E-G). In regulon analysis, EPAS1, SMAD3, and SMAD4 regulons were highly upregulated in alveolar macrophages in AATD (Fig 2H), whereas the RELA, NFkB1, ATF6, and ATF4 regulons were upregulated in AT2s in AATD (Fig 2D). These findings in AT2s are consistent with the known activation of the PERK/ATF4 pathway in ZZ-AATD circulating blood monocytes^3^ and in other AT2 protein misfolding diseases,^29^ in which it has been associated with the local elaboration of inflammatory cytokines and a fibrotic response to injury. Next, to identify cellular stress potentially resulting from transcriptomic derangement in AT2s, we examined expression of the apoptosis mediator cleaved caspase 3. Analysis of peripheral lung tissue by immunostaining for cleaved caspase 3 and pro-SFTPC demonstrated increased frequency of cleaved caspase 3 expression in pro-SFTPC positive cells (17% ± 5.5%, mean ± SD) compared with non-AATD tissue (3.1% ± 3.2% in wild-type COPD and 3.9% ± 3.3% in healthy control participants with no COPD) (Fig 2I-J).

## Discussion

Overall, we found that AAT is expressed in AT2s in addition to alveolar macrophages in primary human lung tissue. AT2s and alveolar macrophages in ZZ-AATD exhibit a distinct transcriptomic signature, including inflammatory pathway and unfolded protein response gene set enrichment. AT2s in ZZ-AATD additionally exhibit evidence of cell stress such as ATF4 regulon enrichment and expression of cleaved caspase 3. Whether observed cellular toxicity results from intracellular polymerization of misfolded Z-AAT protein, as occurs in hepatocytes in AATD, remains to be established. Limitations of this study include the small number of samples from patients with AATD available for sequencing and a lack of tissue representative of earlier stages of disease for analysis. Additional studies will be necessary to identify underlying disease mechanisms responsible for activation of specific pathways within AAT-expressing cells as well as heterogeneity that could exist within the ZZ-AATD population. These findings extend the prevailing paradigm of emphysema pathogenesis in ZZ-AATD. Based on the success of early-phase clinical trials testing the application of small interfering RNA to downregulate Z-AAT expression in hepatocytes,^30^ these data likewise suggest AAT-expressing resident lung cells as logical therapeutic targets for future study.

## Funding/Support

National Institutes of Health (NIH) F30HL147426 (K. M. A.); NIH 1UL1TR001430 (C. M.); NIH R01 HL155821 (E. C.); NIH K08 HL163398 (M. C. B.); NIH KL2TR001879 (S. M. L.); NIH UL1T002345 and R01 HL152968, United States Department of Defense grants W81XWH2010603 and W81XWH2210281, and a grant from the MidAmerica Transplant Association (D. E. B.); and NIH grants U01TR001810, R01DK101501, R01DK117940, R01HL166407, P01HL152953, and P01HL170952 and a grant from the Alpha-1 Foundation (A. A. W.).

## Financial/Nonfinancial Disclosures

The authors have reported to *CHEST* the following: E. C. reports research funding from XVIVO Inc, CareDx, and Pulmocide as well as consulting fees from CSL Behring and United Therapeutics and serves in a leadership position for ISHLT and UNOS and as a consultant for the US Food and Drug Administration. A. A. W. has received research funding from Grifols, Inc. and Beam Therapeutics within the last 3 years for projects distinct from this work and serves as the Scientific Director of the Alpha-1 Foundation. None declared (K. M. A., C. M., M. C. B., S. M. L., M. P. M., P. B., M. G., D. E. B., E. E. M).
